# Bi-functional IgG-lysosomal enzyme fusion proteins for brain drug delivery

**DOI:** 10.1038/s41598-019-55136-4

**Published:** 2019-12-09

**Authors:** Ruben J. Boado, Jeff Zhiqiang Lu, Eric Ka-Wai Hui, Huilan Lin, William M. Pardridge

**Affiliations:** ArmaGen, Inc. Agoura Hills, California, 91301 United States

**Keywords:** Neuroscience, Neurology

## Abstract

Most lysosomal storage disorders affect the central nervous system. However, lysosomal enzymes do not cross the blood-brain barrier (BBB), and intravenous enzyme infusion is not effective for the brain. Lysosomal enzymes can be re-engineered for BBB transport as IgG-enzyme fusion proteins, where the IgG domain is a monoclonal antibody (MAb) against an endogenous BBB receptor/transporter, and which acts as a molecular Trojan horse to deliver the enzyme to brain. However, the problem is retention of high enzyme activity following enzyme fusion to the IgG. The present investigation shows this is possible with a versatile approach that employs fusion of the enzyme to either the IgG heavy chain or light chain using a long flexible linker. The model IgG is a chimeric monoclonal antibody (MAb) against the human insulin receptor (HIR). The enzyme activity of the HIRMAb-enzyme fusion protein is preserved for hexosaminidase A, which is mutated in Tay Sachs disease, for protein palmitoylthioesterase-1, which is mutated in Batten disease type 1, acid sphingomyelinase, which is mutated in Niemann Pick disease type A, and beta galactosidase-1, which is mutated in GM1 gangliosidosis.

## Introduction

Lysosomal storage diseases are inborn errors of metabolism caused by mutations in the gene encoding one of >50 different lysosomal enzymes, and the majority of these inherited diseases adversely affect the central nervous system (CNS)^1^. In most cases, the cDNA encoding the respective lysosomal enzyme has been cloned, and the recombinant protein has been expressed. However, for most disorders, no intravenous (IV) Enzyme Replacement Therapy (ERT) has been developed because (a) the primary clinical manifestations of the disease are CNS, and (b) the recombinant enzyme does not cross the blood-brain barrier (BBB). For example, Tay Sachs disease (TSD)^2^ is caused by mutations in the gene encoding the hexosaminidase A (HEXA) enzyme^3^, but HEXA does not cross the BBB^4^. Batten type 1 disease, also known as Ceroid Lipofuscinosis type 1 (CLN1) disease^5^, is caused by mutations in the gene encoding protein palmitoylthioesterase-1 (PPT1)^6^, but PPT1 does not cross the BBB^7^. Niemann Pick disease type A (NPDA)^8^ is caused by mutations in the gene encoding acid sphingomyelinase (ASM)^9^, also known as sphingomyelin phosphodiesterase-1 (SMPD1), but ASM does not cross the BBB^10^. GM1 gangliosidois (GM1G)^11^ is caused by mutations in the gene encoding beta galactosidase-1 (GLB1)^12^, but GLB1 does not cross the BBB^13^.

The lysosomal enzymes can be re-engineered to enable BBB transport with the genetic engineering of an IgG-lysosomal enzyme fusion protein, where the IgG domain is a monoclonal antibody (MAb) directed against an endogenous BBB receptor-mediated transport system, such as the insulin receptor or the transferrin receptor^14^. The IgG domain acts as a molecular Trojan horse to ferry the enzyme across the BBB and into brain cells. Typically, the lysosomal enzyme is genetically fused to the carboxyl terminus (CT) of the heavy chain (HC) of the IgG with a short, e.g. 3-amino acid (AA) linker^14^.

The challenge in engineering IgG-lysosomal enzyme fusion proteins is the retention of high lysosomal enzyme activity following fusion of the enzyme to the IgG. For example, fusion of the beta glucuronidase (GUSB) enzyme to the CT of the HC of an IgG resulted in >95% loss of enzyme activity^15^. Fusion of the enzyme to the amino terminus (NT) of the IgG resulted in retention of high enzyme activity, but this format caused a >95% loss of binding of the IgG to the target BBB receptor^15^, owing to steric hindrance caused by fusion of the enzyme to the IgG NT domain, which is contiguous with the complementarity determining regions (CDR) of the IgG. However, enzyme activity may be retained following fusion to an IgG with the use of alternative genetic engineering strategies.

The purpose of the present investigation is to test the versatility of the IgG-enzyme platform with respect to alternative approaches toward the engineering of IgG-enzyme fusion proteins for lysosomal enzymes such as HEXA, PPT1, ASM, and GLB1. The engineering of the IgG-enzyme fusion protein may involve (a) fusion of the enzyme to the CT of either the IgG heavy chain (HC) or the light chain (LC), or (b) the use of either a short linker (SL), or a long linker (LL) between the IgG chain and the enzyme. A long flexible linker provides spatial separation between the IgG and enzyme domains of the fusion protein. A longer linker may be more immunogenic than a short linker. Therefore, the long linker should be comprised of a linker that is both flexible and derived from an existing human protein sequence. These properties are satisfied with the use of a linker derived from the hinge region of human IgG3, which is longer and more flexible than the hinge region of any other human IgG subclass^16^. The model IgG domain is a chimeric MAb against the human insulin receptor (HIR), designated the HIRMAb^14^.

This study shows that HEXA, PPT1, ASM, or GLB1 can be fused to the CT of either the HC or LC of the HIRMAb, and enzyme activity is comparable to the native enzyme with the use of a long flexible linker.

## Results

### Nomenclature

The fusion proteins are designated as HIRMAb-LC-LL-HEXA, HIRMAb-HC-SL-PPT1, HIRMAb-HC-LL-PPT1, HIRMAb-LC-LL-ASM, HIRMAb-HC-SL-GLB1, and HIRMAb-HC-LL-GLB1, where the LC or HC designation indicates if the enzyme was fused to the CT of the heavy chain or the light chain, respectively, and the SL and LL designation refers to the presence of either the 3-AA short linker, or the 31-AA long linker, respectively.

### Western blotting

**Figure 1:**
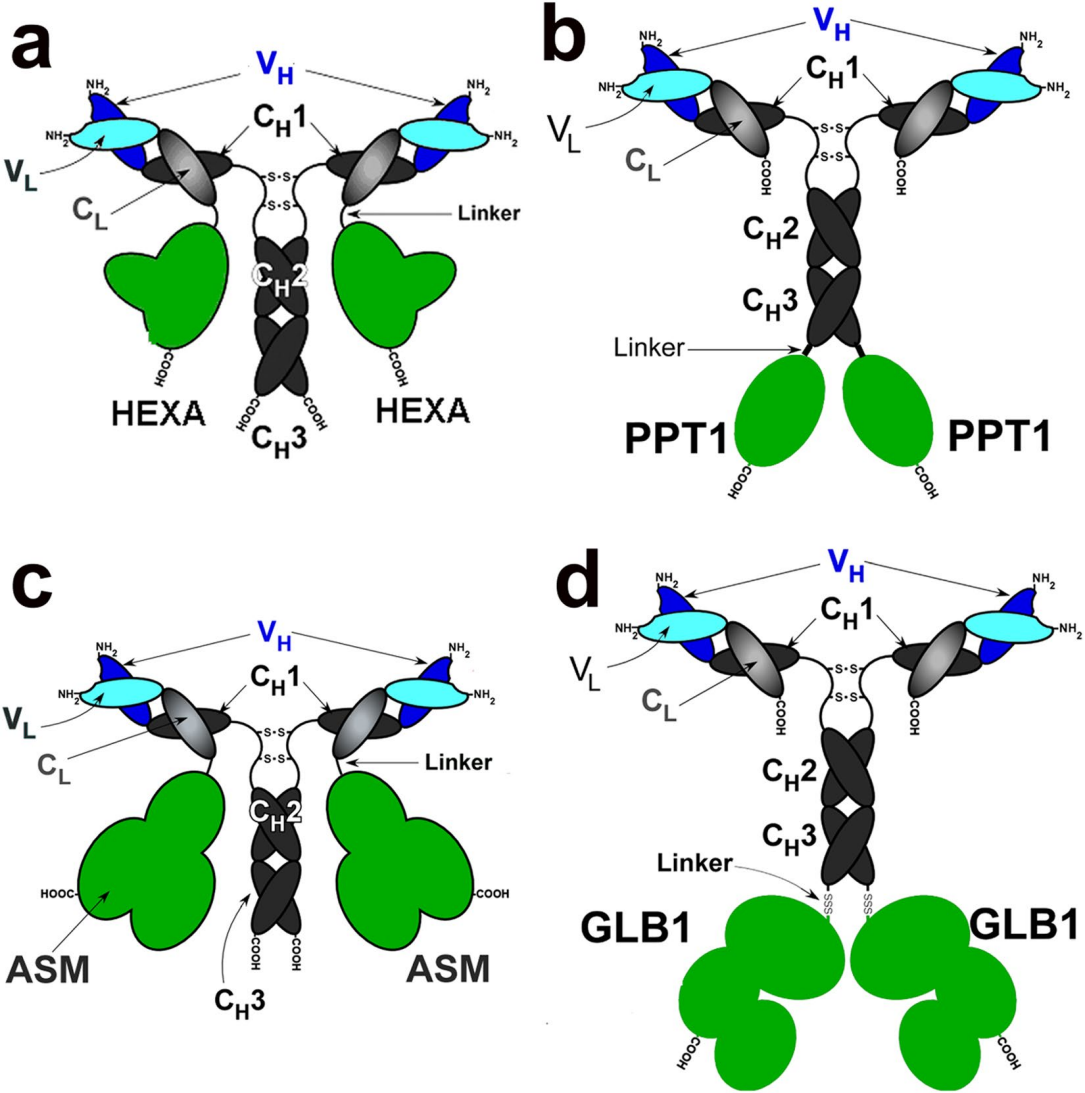
(**a**) Structure of HIRMAb-LC-LL-HEXA, where the HEXA enzyme is fused to the CT of the light chain (LC) of the HIRMAb via a 31-amino acid long linker (LL). (**b**) Structure of HIRMAb-HC-LL-PPT1, where the PPT1 enzyme is fused to the CT of the heavy chain (HC) of the HIRMAb via the LL linker. (**c**) Structure of HIRMAb-LC-LL-ASM, where the ASM enzyme is fused to the CT of the LC of the HIRMAb via the LL linker. (**d**) Structure of HIRMAb-HC-LL-GLB1, where the GLB1 enzyme is fused to the CT of the HC of the HIRMAb via the LL linker. V_H_ = variable region of HC; V_L_ = variable region of LC; C_L_ = constant region of LC; C_H_1, C_H_2, and C_H_3 are domains of the constant region of the HC.

**Figure 2:**
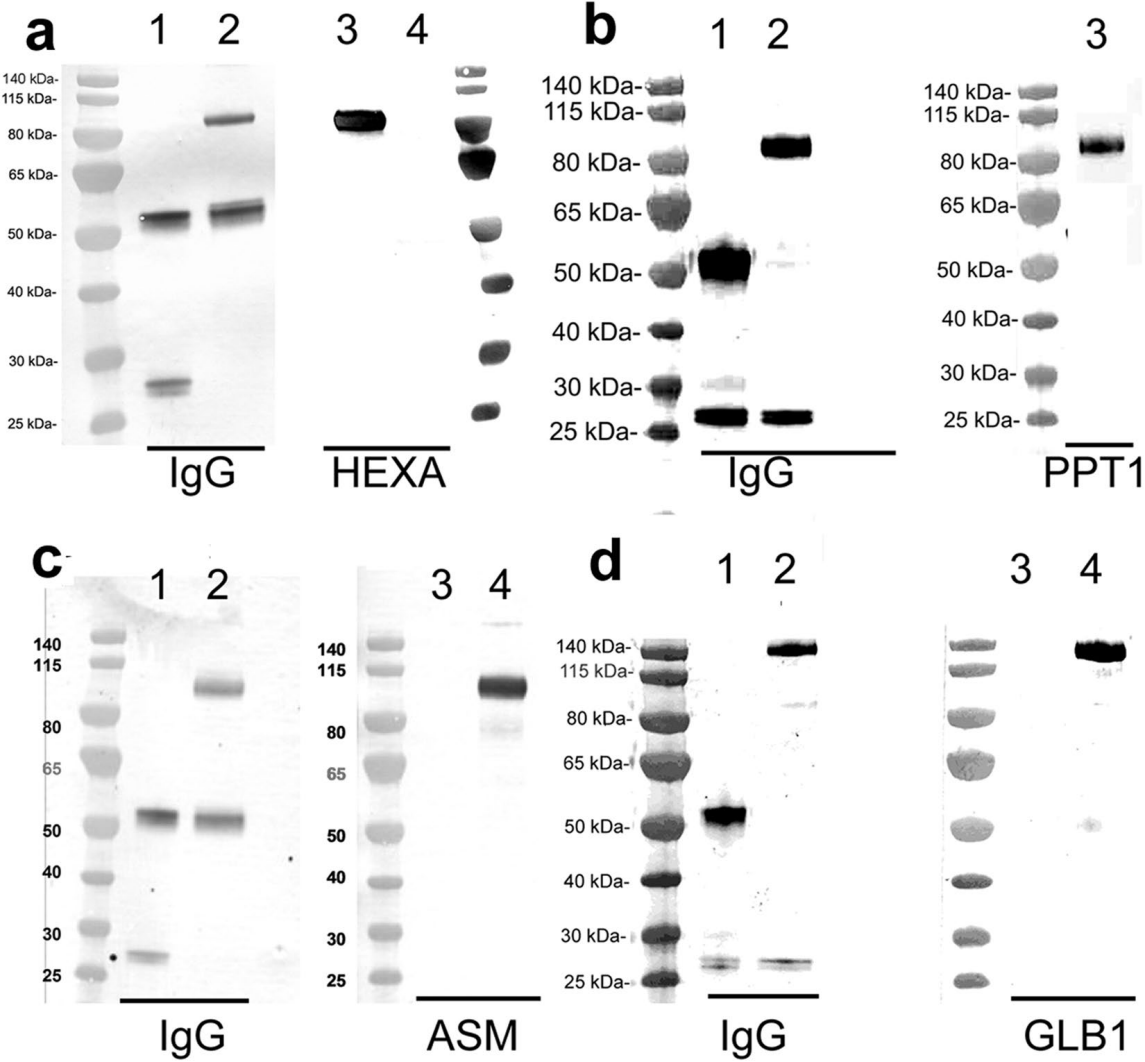
(**a**) Western blot (WB) of the HIRMAb (lanes 1,4) and the HIRMAb-LC-LL-HEXA fusion protein (lanes 2,3) probed with a primary antibody against either human IgG (left panel, lanes 1,2) or against human HEXA (right panel, lanes, 3,4). (**b**) WB of the HIRMAb (lane 1) and the HIRMAb-HC-LL-PPT1 fusion protein (lanes 2,3) probed with a primary antibody against either human IgG (left panel, lanes 1,2) or against human PPT1 (right panel, lane 3). (**c**) WB of the HIRMAb (lanes 1,3) and the HIRMAb-LC-LL-ASM fusion protein (lanes 2,4) probed with a primary antibody against either human IgG (left panel, lanes 1,2) or against human ASM (right panel, lanes 3,4). (**d**) WB of the HIRMAb (lanes 1,3) and the HIRMAb-HC-LL-GLB1 fusion protein (lanes 2,4 probed with a primary antibody against either human IgG (left panel, lanes 1,2) or against human GLB1 (right panel, lanes 3,4).

The structures of the 4 IgG-enzyme fusion proteins are shown in Fig. 1. The HEXA and the ASM are fused to the CT of the light chain of the HIRMAb, and PPT1 and GLB1 are fused to the CT of the heavy chain of the HIRMAb. Following protein A affinity chromatography, each fusion protein migrated on reducing SDS-PAGE with a single heavy chain (HC) and single light chain (LC). The Western blots (WB) for the 4 fusion proteins are shown in Fig. 2. For the HEXA fusion protein, the anti-human IgG WB detected a 56 kDa HC for both the HIRMAb (lane 1) and the HIRMAb-LC-LL-HEXA (lane 2), and detected a 26 kDa LC for the HIRMAb (lane 1) and a 102 kDa LC for the HIRMAb-LC-LL-HEXA (lane 2) (Fig. 2a, left panel). For the HEXA fusion protein, the anti-HEXA WB detected the 102 kDa HIRMAb-LC-LL-HEXA (lane 3) and showed no reaction with the HIRMAb (lane 4) (Fig. 2a, right panel). For the PPT1 fusion protein, the anti-human IgG WB detected a 56 kDa HC for the HIRMAb and a 99 kDa HC for the HIRMAb-HC-LL-PPT1, and detected a 26 kDa LC for both the HIRMAb (lane 1) and HIRMAb-HC-LL-PPT1 (lane 2) (Fig. 2b, left panel). For the PPT1 fusion protein, the anti-PPT1 WB detected only the 99 kDa HIRMAb-HCC-LL-PPT1 (lane 3) (Fig. 2b, right panel). For the ASM fusion protein, the anti-human IgG WB detected a 56 kDa HC for both the HIRMAb and the HIRMAb-LC-LL-ASM, and detected 26 kDa LC for the HIRMAb (lane 1) and a 105 kDa LC for the HIRMAb-LC-LL-ASM (lane 2) (Fig. 2c, left panel). For the ASM fusion protein, the anti-ASM WB detected the 105 kDa HIRMAb-LC-LL-ASM (lane 4) and showed no reaction with the HIRMAb (lane 3) (Fig. 2c, right panel). For the GLB1 fusion protein, the anti-human IgG WB detected a 56 kDa HC for the HIRMAb and a 140 kDa HC for the HIRMAb-HC-LL-GLB1, and detected a 26 kDa LC for both the HIRMAb (lane 1) and HIRMAb-HC-LL-GLB1 (lane 2) (Fig. 2d, left panel). For the GLB1 fusion protein, the anti-GLB1 WB showed no reaction with the HIRMAb (lane 3), but detected the 140 kDa HIRMAb-HC-LL-GLB1 (lane 4) (Fig. 2d, right panel). The MWs estimated from the WB are given in Table 1 for the HC, the LC, and the tetramer, in comparison with the MWs of the non-glycosylated fusion proteins estimated from the amino acid sequence.

**Table 1:** Molecular weights of fusion protein chains and tetramers computed from either the predicted amino acid (AA) sequence or by Western blotting (Fig. 2).

Fusion protein	Chain/tetramer	Molecular weight	
		AA sequence	Western blot
HIRMAb LC-LL- HEXA	Heavy chain	48,611	56,000
	Light chain	84,881	102,000
	tetramer	266,984	316,000
HIRMAb-HC-LL-PPT1	Heavy chain	82,869	99,000
	Light chain	23,402	26,000
	tetramer	212,542	250,000
HIRMAb-LC-LL-ASM	Heavy chain	48,611	56,000
	Light chain	89,787	105,000
	tetramer	276,796	322,000
HIRMAb-HC-LL-GLB1	Heavy chain	125,176	140,0000
	Light chain	23,402	26,000
	tetramer	297,156	332,000

### HIR binding

**Figure 3:**
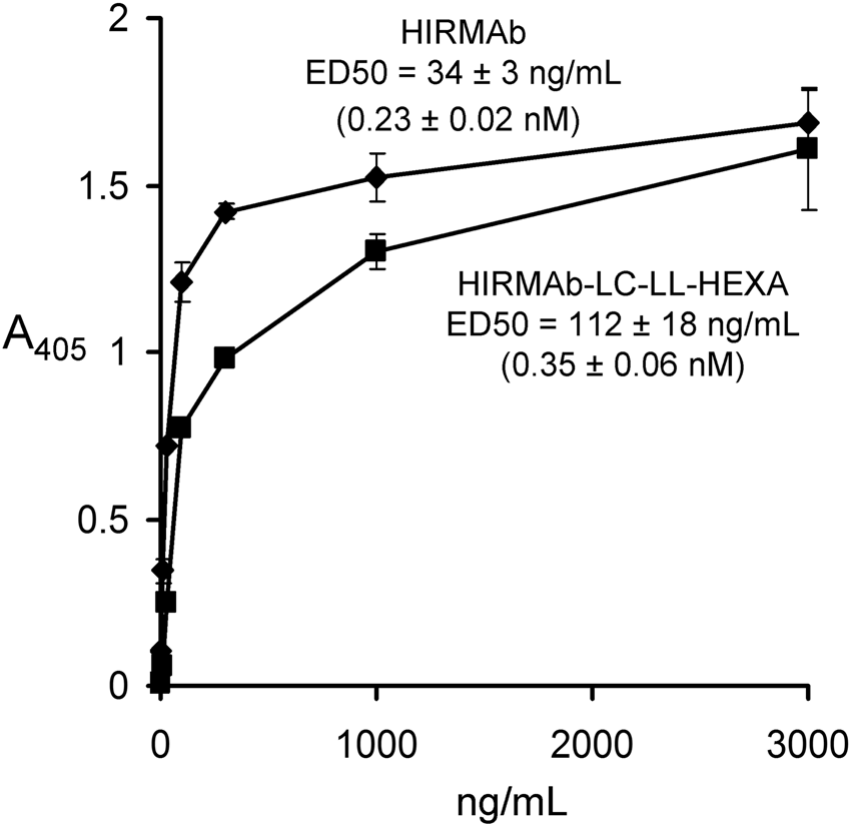
Binding of HIRMAb or HIRMAb-LC-LL-HEXA as determined by ELISA where the capture agent is the extracellular domain of the recombinant human insulin receptor (HIR) and the detector reagent is a conjugate of alkaline phosphatase and a goat anti-human IgG-Fc antibody. The ED50 was determined by non-linear regression analysis.

The binding of the HIRMAb and the HIRMAb-LC-LL-HEXA fusion protein to the HIR is shown in Fig. 3. The HIR binding of the respective HIRMAb-enzyme fusion proteins were determined in 4 separate ELISAs, using the HIRMAb alone as control, and these ED50 values are listed in Table 2. The ED50 was initially measured as ng/mL, and was converted to nM, based on the MW of the tetramer determined by Western blotting (Table 1). The ED50 of the HIRMAb alone varied within a narrow range of 0.23–0.32 nM (Table 1). The ED50 of 3 of the HIRMAb-enzyme fusion proteins also varied within a narrow range of 0.35–0.38 nM, the one exception being the ED50, 0.93 nM, of the HIRMAb-ASM fusion protein, which still represents a high affinity for the HIR.

**Table 2:** Fusion protein binding to human insulin receptor (HIR) as determined by ELISA using the HIR extracellular domain as capture agent. ED50 values are mean ± SE, as determined by non-linear regression analysis of saturation curves such as shown in Fig. 3.

Assay	Protein	HIR ED50 (nM)
1	HIRMAb	0.23 ± 0.02
	HIRMAb-LC-LL-HEXA	0.35 ± 0.06
2	HIRMAb	0.26 ± 0.04
	HIRMAb-HC-LL-PPT1	0.38 ± 0.11
3	HIRMAb	0.32 ± 0.11
	HIRMAb-LC-LL-ASM	0.93 ± 0.12
4	HIRMAb	0.25 ± 0.04
	HIRMAb-HC-LL-GLB1	0.36 ± 0.05

### Enzyme activity

The enzyme activity of each fusion protein was measured with flurometric assays and enzyme-specific substrates (Table 3), and the specific activity for each fusion protein is given in Table 3. Using the 4-MUG substrate, the HEXA activity of HIRMAb-LC-LL-HEXA was comparable to recombinant HEXA. The ratio of HEXA enzyme activity of the HIRMAb-LC-LL-HEXA fusion protein with the 4-MUG substrate, relative to the 4-MUGS substrate, was 1.5 (Table 3). Initially, the HIRMAb-HC-PPT1 fusion protein was engineered with the short linker (SL), but this HIRMAb-HC-SL-PPT1 fusion protein demonstrated low PPT1 enzyme activity (Table 3). Subsequently, this fusion protein was re-engineered with the long linker (LL), and the HIRMAb-HC-LL-PPT1 fusion protein exhibited high PPT1 enzyme activity (Table 3). The HIRMAb-LC-LL-ASM fusion protein showed high enzyme activity against the HMU-PC substrate (Table 3). Initially, the HIRMAb-HC-GLB1 fusion protein was engineered with the SL short linker, but this HIRMAb-HC-SL-GLB1 fusion protein demonstrated reduced GLB1 enzyme activity compared to human recombinant GLB1 (Table 3). Subsequently, this fusion protein was re-engineered with the long LL linker, and the HIRMAb-HC-LL-GLB1 fusion protein exhibited high GLB1 enzyme activity (Table 3).

**Table 3:** Lysosomal enzyme activity of fusion proteins as determined with enzyme-specific substrates and enzymatic fluorometric assays.

Substrate	IgG-enzyme or enzyme	Enzyme activity	Units of activity
4-methylumbelliferyl-2-acetamido-2-deoxy-β-D-glucopyranoside (4-MUG)	HIRMAb-LC- LL-HEXA	2,464 ± 109	mU/mg protein
	HEXA	2,557 ± 187	
4-Methylumbelliferyl-7-(6-sulfo-2-acetamido-2-deoxy-β-D-glucopyranoside (4-MUGS)	HIRMAb-LC- LL-HEXA	1,636 ± 203	
4-methylumbelliferyl 6-thio-palmitate-β-D-glucopyranoside (Mu-6S-Palm-beta-Glc)	HIRMAb-HC- SL-PPT1	53 ± 15	mU/mg protein
	HIRMAb-HC- LL-PPT1	1742 ± 75	
6-hexadecanoylamino-4-methylumbelliferyl phosphocholine (HMU-PC)	HIRMAb-LC- LL-ASM	902 ± 41	mU/mg protein
4-methylumbelliferyl β-D-galactopyranoside (MUGP)	HIRMAb-HC- SL-GLB1	49,000 ± 12,000	units/mg protein
	HIRMAb-HC- LL-GLB1	171,000 ± 23,000	
	GLB1	183,000 ± 56,000	

Enzyme activity units are: 1 milliunit (mU) = 1 nmol per minute for HEXA; 1 mU = 1 nmol per minute for PPT1; 1 mU = 1 nmol per minute for ASM; 1 unit = 1 nmol per hour for GLB1. Values are mean ± SE (N = 3 replicates).

## Discussion

This investigation describes the genetic engineering of 4 bi-functional IgG-lysosomal enzyme fusion proteins, where the IgG domain is the chimeric HIRMAb and the lysosomal enzyme domain is comprised of human HEXA, PPT1, ASM, or GLB1. Each of these lysosomal enzymes are mutated in TSD, CLN1, NPDA, or GM1G, respectively^2,5,8,11^. All 4 of these lysosomal storage disorders have severe CNS clinical manifestations^2,5,8,11^ and conventional ERT is not possible, because the enzymes do not cross the BBB. Prior work has shown that the HIRMAb does cross the BBB via RMT on the endogenous BBB insulin receptor, and delivers a fused lysosomal enzyme to the primate brain with a brain uptake of 1% injected dose (ID)/brain^17,18^. As discussed below, this level of brain uptake produces therapeutic levels of lysosomal enzyme activity in the brain, providing the activity of the respective lysosomal enzyme is retained following genetic fusion of the enzyme to the HIRMAb. IgG-lysosomal enzymes are triaged to the lysosomal compartment^17^, and reduce lysosomal inclusion bodies in both peripheral organs and the brain following systemic administration in mouse models of lysosomal storage disease^19,20^. The HIRMAb-enzyme fusion proteins have dual receptor specificity and, in addition to binding the insulin receptor, also bind the mannose 6-phosphate (M6P) receptor (M6PR)^18^. The HIRMAb-enzyme fusion proteins are targeted to somatic tissues via the M6PR and to the CNS via the BBB insulin receptor^18^. HEXA, PPT1, ASM, and GLB1 all incorporate M6P^21^. The HIRMAb-iduronidase (IDUA) fusion protein, valanafusp alpha, has an excellent safety profile in humans over the course of 52 weeks of treatment of Mucopolysaccharidosis (MPS) Type I (MPSI), and stabilizes both somatic and CNS function in children with MPSI at weekly intravenous infusion doses of 1–6 mg/kg^22^.

The lysosomal enzyme can be fused to the C-terminus of either the heavy chain (HC) or the light chain (LC) of the IgG domain. Fusion to the HC places the enzyme in a dimeric configuration, whereas fusion to the LC places the enzyme in a more flexible, monovalent configuration. Enzymes such as PPT1^23^ or GLB1^24^ form homo-dimers, and these enzymes were fused to the C-terminus of the HC (Fig. 1b,d). Conversely, enzymes such as HEXA or ASM form hetero-dimers with activator proteins, such as GM2 activator protein^25^ or saposin C^26^, respectively, and these enzymes were fused to the C-terminus of the LC (Fig. 1a,c).

TSD is a severe childhood form of neurodegeneration of the brain and spinal cord leading to psychomotor retardation, seizures, deafness, and death in early childhood^2^. The cDNA encoding human HEXA was published in 1985^3^. HEXA does not cross the BBB^4^, so intravenous ERT of TSD is not possible. The HIRMAb-HEXA fusion protein retains both high affinity binding to the HIR (Table 2) and HEXA enzyme activity comparable to recombinant HEXA (Table 3). The normal HEXA enzyme activity in brain, as determined with the same substrate, 4-MUGS, used in this study, is 10.8 nmol/hour/mgp^27^, which is equivalent to 18 milliunits/gram, where 1 mU = 1 nmol/min, and 1 gram brain is equal to 100 mg protein (mgp)^28^. The HEXA enzyme activity administered with an ID of 3 mg/kg of the HIRMAb-LC-LL-HEXA fusion protein, in a 50 kg human, is equivalent to 250,000 milliunits, given the specific activity of 1636 mU/mg for this fusion protein (Table 3). With a brain uptake of 1% ID/brain, and a 1000 g human brain, the HEXA activity delivered to brain is 2500 milliunits/brain, or 2.5 milliunits/gram, which is 23% of normal endogenous HEXA activity in brain. The replacement of only 1–2% of endogenous lysosomal enzyme activity is sufficient to reverse the course of the disease^29^. The replacement of 1–5% of HEXB enzyme activity in the Sandhoff mouse is sufficient to reverse ganglioside storage and to prolong life^30^.

CLN1 is a severe form of Batten disease associated with neurodegeneration, muscle weakness, blindness, seizure and early death in childhood^5^. The cDNA encoding human PPT1 was published in 1994^6^. Human PPT1 has been expressed in CHO cells as a 34 kDa protein^31^, with an enzyme activity of 15 units/mg^32^, using the same Mu-6S-Palm-betaGlc substrate used in this study (Table 3). The PPT1 specific activity of the HIRMAb-HC-LL-PPT1 fusion protein is 1.7 units/mg (Table 3). However, the MW of the fusion protein half-tetramer, 125 kDa (Table 1), is 4-fold greater than the MW of the PPT1 monomer; therefore, on a molar basis, the PPT1 enzyme activity is 46% of the activity of recombinant human PPT1. PPT1 does not cross the BBB^7^, and intravenous ERT of the brain with recombinant PPT1 is not possible. The PPT1 enzyme has been delivered to brain of the CLN1 mouse via intra-cerebroventricular (ICV) injection^7^. However, ICV administration only delivers enzyme to the surface of the brain, as demonstrated in primates following the ICV infusion of very high doses of the lysosomal enzyme^33^. Treatment of the entire parenchyma of brain is possible with transvascular delivery of PPT1 across the BBB. The normal brain PPT1 enzyme activity is 70 nmol/hour/mgp using the Mu-6S-Palm-betaGlc substrate^34^, which is equal to 116 mU/gram, where 1 mU = 1 nmol/min, and 1 gram brain is equal to 100 mgp. The PPT1 enzyme activity administered with an ID of 3 mg/kg of the HIRMAb-HC-LL-PPT1 fusion protein, in a 50 kg human, is equivalent to an ID of 260,000 milliunits, given the specific activity of 1742 mU/mg for this fusion protein (Table 3). With a brain uptake of 1% ID/brain, and a 1000 g human brain, the PPT1 activity delivered to brain is 2600 milliunits/brain, or 2.6 milliunits/gram, which is 2.2% of normal endogenous PPT1 activity in brain. A brain PPT1 activity as low as 0.5% of endogenous activity is sufficient to reverse the neuropathology of CLN disease^35^.

NPDA is a neurodegenerative condition leading to muscle weakness, mental retardation, seizures, and early death in childhood^8^. The cDNA encoding human ASM was published in 1989^9^. Estimates of the enzyme activity of recombinant ASM with the HMU-PC substrate used in this study are not readily available, although the activity of 902 mU/mg for the HIRMAb-LC-LL-ASM fusion protein (Table 3) is comparable to the activity of recombinant ASM, 1700 mU/mg, as determined with a similar substrate, 2-N-hexadecanoylamino-4-nitrophenylphosphorylcholine (R&D Systems, Minneapolis, MN). The MW of the half-tetramer of the HIRMAb-LC-LL-ASM fusion protein, 161 kDa, is nearly 2-fold greater the MW of recombinant human ASM, 75 kDa^36^. Therefore, the ASM activity of the HIRMAb-LC-LL-ASM fusion protein is comparable to recombinant ASM. The ASM activity in brain with the HMU-PC substrate is not readily available. However, intracerebral injection of adeno-associated virus encoding ASM produces ASM brain at levels of 0.4–1.9 ug/gram, and this reverses neuropathology in the ASM knockout mouse^37^. The administration of 3 mg/kg of the HIRMAb-LC-LL-ASM fusion protein in a 50 kg human is expected to produce a brain concentration of the fusion protein of 1% ID/brain, or 1500 ug/brain or 1.5 ug/gram brain, which is a therapeutic enzyme level in the brain of the NPDA mouse^37^.

GM1G infants are afflicted with neurodegeneration, seizures, ataxia, and blindness followed by early death in childhood^11^. The cDNA encoding human GLB1 was published in 1988^12^. Recombinant GLB1 has been expressed in CHO cells with a specific activity of 240,000 units/mg^38^, using the MUGP substrate, and this value is not significantly different from the specific activity of commercially available recombinant human GLB1 (Table 3). The MW of CHO derived GLB1 is 88 kDa^39^, which is nearly 2-fold lower than the MW of the half-tetramer of the HIRMAb-HC-LL-GLB1 fusion protein (Table 3). The endogenous GLB1 enzyme activity in brain, using the MUGP substrate, is 57 units/mgp^40^, which is comparable to 5700 units/gram brain, assuming 1 gram brain is 100 mgp. The GLB1 enzyme activity administered with an ID of 3 mg/kg of the HIRMAb-HC-LL-GLB1 fusion protein, in a 50 kg human, is equivalent to 25.6 million units, given the specific activity of 171,000 units/mg (Table 3). With a brain uptake of 1% ID/brain, and a 1000 g human brain, the GLB1 activity delivered to brain is 256,000 units/brain, or 256 units/gram, which is 4.5% of normal endogenous GLB1 activity in brain. This level of GLB1 activity in brain may be therapeutic as GLB1 activity at the 10% of normal level is sufficient to lower brain GM1 ganglioside^41^. Higher levels of GLB1 enzyme activity are possible with injection doses >3 mg/kg.

The immunogenicity, or effector function, of HIRMAb-enzyme fusion proteins has been examined in humans following a year of chronic administration, and the immunogenicity of the fusion protein is no greater than the immunogenicity of the enzyme alone, and no clinical signs of effector function were observed^22^. The insertion of the IgG3 hinge region in engineered antibodies does not increase effector function^42^.

In summary, the present investigation shows that a diverse group of lysosomal enzymes can be successfully fused to either the LC or the HC of the HIRMAb and that high enzyme activity is retained with the use of 31-AA long linker between the IgG and enzyme domains. This linker is expected to be stable *in vivo* and not to be immunogenic as the sequence is derived from the human IgG3 hinge region. Fusion of the lysosomal enzyme to a BBB molecular Trojan horse, such as the HIRMAb, enables the intravenous ERT of the brain in these serious childhood inborn errors of metabolism.

## Methods

### Genetic engineering and production of HIRMAb-lysosomal enzyme fusion proteins

The HEXA domain corresponded to Leu-23 to Thr-529 of human HEXA (NP_000511); the PPT1 domain corresponded to Asp-28 to Gly-306 of human PPT1 (NP_000301); the ASM domain corresponded to His-62 to Pro-628 of human ASM (NP_000534); the GLB1 domain corresponded to Leu-24 to Val-677 of human GLB1 (NP_000395). The HC of the HIRMAb is comprised of a 113 AA variable region of the heavy chain (VH) followed by the constant (C)-region of human IgG1; the LC of the HIRMAb is comprised of a 108 AA variable region of the light chain (VL) followed by the C-region of the human kappa light chain. The linker joining the CT of either the HC or LC was either a short, (Ser)3 linker or a long 31-AA linker. The 31 AA linker includes 25 AA from the human IgG3 hinge region, and is derived from the 12 AA of the upper hinge region, followed by 5 AA of the first part of the core hinge region, followed by 8 AA of the lower hinge region, and is flanked by a Ser-Ser-Ser sequence on the amino terminus and a Ser-Ser-Ser sequence on the carboxyl terminus of the linker, as discussed previously^43^. The 2 cysteine residues of the first part of the core hinge region are mutated to serine residues, so as to eliminate disulfide bonding. The first Leu of the lower hinge is mutated to Phe to eliminate complement fixation^42^. A synthetic gene encoding the lysosomal enzyme and linker was produced at GenScript (Piscataway, NJ), and subcloned into a HIRMAb HC or LC expression plasmid under the influence of a hybrid cytomegalovirus promoter and the bovine growth hormone polyA sequence, which also contained an expression cassette encoding for dihydrofolate reductase to allow of selection of stably transected Chinese hamster ovary (CHO) lines with methotrexate. The genetic engineering of all expression plasmids was confirmed by agarose gel electrophoresis following digestion with specific restriction endonucleases, and bidirectional DNA sequencing using custom primers. The molecular weights (MW) of the non-glycosylated fusion protein were predicted from the amino acid sequence, and the MWs for the HC, the LC, and the tetramer are given in Table 1.

COS cells were transiently transfected with Lipofectamine 2000. Stably transfected CHO lines in serum free medium were cloned following dual electroporation with the HC and LC expression plasmids. CHO lines were subjected to 1 cell/well dilutional cloning, and the IgG expression ranged from 10–100 mg/L in shake flasks with a cell density of approximately 10^5^–10^6^ cells/mL.

CHO cells were cultured in shake flasks, and the IgG-enzyme fusion protein was purified from this conditioned medium by protein A affinity chromatography. The purity and identity of each fusion protein was examined by reducing sodium dodecylsulfate polyacrylamide gel electrophoresis (SDS-PAGE), and Western blotting (WB), respectively. The primary antibody for the human IgG WB was a goat anti-human IgG (H + L) antibody (Vector Labs, Burlingame, CA), and the secondary antibody was a biotinylated horse anti-goat IgG (Vector). The primary antibody for the enzyme WB was a mouse anti-human HEXA, a goat anti-human ASM, and a mouse anti-human GLB1 (all from R&D Systems, Minneapolis, MN), and a mouse anti-human PPT1 antibody (Abcam, Cambridge, MA), and the secondary antibody was either a biotinylated horse anti-mouse IgG or a biotinylated horse anti-goat IgG (Vector Labs).

### Human insulin receptor binding assay

The binding avidity of the fusion protein for the HIR was determined by ELISA. The capture agent is the HIR extracellular domain from R&D Systems (Minneapolis, MN), and the detector agent is a conjugate of alkaline phosphatase and a goat anti-human IgG-Fc antibody (Abcam, Carmbridge, MA). The concentration of either HIRMAb alone, or HIRMAb-enzyme fusion protein, that caused 50% of maximal binding, the EC50, was determined by non-linear regression analysis by fitting the data to: Absorbance = (Amax·S)/(EC50 + S), where Amax is the maximal absorbance and S = the concentration of IgG or fusion protein. The EC50 was estimated with the units of ng/mL, and converted to units of nM, based on the molecular weight (MW) as determined by migration in reducing SDS-PAGE.

### Lysosomal enzyme activity assays

The HEXA flurometric enzymatic assay was performed as described using either 0.5 mM 4-methylumbelliferyl-N-acetyl-β-D-glucosaminide (4-MUG, Sigma-Aldrich, St Louis, MO) or 0.5 mM 4-Methylumbelliferyl-7-(6-sulfo-2-acetamido-2-deoxy-β-D-glucopyranoside) (4-MUGS, Toronto Research Chemicals) as the assay substrate^44^. The assay buffer was 100 mM Na citrate, 250 mM NaCl, pH = 4.5; incubations were performed for 20 min at 37 C and terminated with alkaline stop solution; enzyme activity is defined as 1 milliunit = 1 nmol/min. Recombinant human HEXA was obtained from R&D Systems (Minneapolis, MN). The PPT1 fluorometric enzymatic assay was performed as described using 0.64 mM 4-methylumbelliferyl 6-thio-palmitate-β-D-glucopyranoside (Mu-6S-Palm-beta-Glc) as assay substrate^45^, and McIlvaine’s phosphate/citrate buffer at pH = 4.0/15 mM dithiothreitol/0.375% Triton X-100, and 5 units/mL of beta-glucosidase (Sigma G4511, St Louis, Mo) as assay buffer; incubations were performed for 60 min at 37 C and terminated with alkaline stop solution; enzyme activity is defined as 1 milliunit = 1 nmol/min. The ASM fluorometric enzymatic assay was performed as described using 0.64 mM 6-hexadecanoylamino-4-methylumbelliferyl phosphorylcholine (HMU-PC, Toronto Research Chemicals) as assay substrate^46^, and 0.1 M sodium acetate/pH = 5.2/0.2% sodium taurocholate as assay buffer; incubations were performed for 60 min at 37 C and terminated with alkaline stop solution; enzyme activity is defined as 1 milliunit = 1 nmol/min. The GLB1 fluorometric enzymatic assay was performed as described using 0.64 mM 4*-*Methylumbelliferyl β*-*D-glucopyranoside (MUGP, Sigma) as assay substrate^39^, and 50 mM Na citrate, pH = 3.5 as assay buffer; incubations were performed for 20 min at 37 C and terminated with alkaline stop solution; enzyme activity is defined as 1 unit = 1 nmol/hour. Recombinant human GLB1 from CHO cells was obtained from R&D Systems. For each of the 4 assays, a standard curve was produced with 0.01 to 3 nmol/tube of 4-methylumbelliferone (4-MU, Sigma-Aldrich). All assays were linear with respect to amount of enzyme added per tube in the range of 10 to 300 ng/tube.
